# Understanding Brain Mechanisms of Reactive Aggression

**DOI:** 10.1007/s11920-020-01208-6

**Published:** 2020-11-12

**Authors:** Katja Bertsch, Julian Florange, Sabine C. Herpertz

**Affiliations:** 1 Department of General Psychiatry, Center for Psychosocial Medicine, Medical Faculty, Heidelberg University, Heidelberg, Germany; 2 Department of Psychology, Ludwig-Maximilians-University, Munich, Germany

**Keywords:** Social threat, Provocation, Frustration, Threat sensitivity, Cognitive control, Emotion regulation, Inhibitory control, Frustrative non-reward

## Abstract

**Purpose of Review:**

To review the current literature on biobehavioral mechanisms involved in reactive aggression in a transdiagnostic approach.

**Recent Findings:**

Aggressive reactions are closely related to activations in the brain’s threat circuitry. They occur in response to social threat that is experienced as inescapable, which, in turn, facilitates angry approach rather than fearful avoidance. Provocation-induced aggression is strongly associated with anger and deficits in cognitive control including emotion regulation and inhibitory control. Furthermore, the brain’s reward system plays a particular role in anger-related, tit-for-tat-like retaliatory aggression in response to frustration. More research is needed to further disentangle specific brain responses to social threat, provocation, and frustration.

**Summary:**

A better understanding of the psychological and neurobiological mechanisms involved in reactive aggression may pave the way for specific mechanism-based treatments, involving biological or psychotherapeutic approaches or a combination of the two.

**Supplementary Information:**

The online version contains supplementary material available at 10.1007/s11920-020-01208-6.

## Introduction

Aggression is an evolutionarily highly conserved behavior directed toward another individual with the intent to cause harm [1]. Reactive aggression is commonly defined as a response to social threat, provocation, or frustration, and is strongly associated with anger [2, 3]. Since increased reactive aggression is found in various mental disorders, it might be better regarded and explained as a transdiagnostic phenomenon. Reactive aggression is related to the following biobehavioral mechanisms: increased threat sensitivity [4] and frustrative non-reward [5, 6] as activating conditions as well as poor cognitive control as a regulatory condition [4, 7]. Within the following three sections, we systematically summarize studies on reactive aggression from a transdiagnostic perspective by focusing on brain data that are associated with the three biobehavioral mechanisms of reactive aggression in healthy subjects and in clinical phenotypes associated with high reactive aggression. Since social threat, provocation, and frustration have been identified as the most important situational triggers for reactive aggression, we attempt to describe, and where possible disentangle, how the three biobehavioral mechanisms are related to these situations. However, it should be noted that a one by one assignment of the situational triggers and the biobehavioral mechanisms is neither intended nor possible. While explanations of the most important constructs and prevailing experimental paradigms that have been applied are included in the text, details about study designs, samples, sample sizes, and paradigms are provided in Table 1 (sorted by first author and year of publication). As can be seen in this table, we have selected studies published between 2013 and 2020, which included structural or functional magnetic resonance imaging (MRI), a measure for aggression, and a sample of ideally at least 30 participants (data that are based on smaller samples were only included if of particular relevance or if independent replication was available; for these studies, *N* is reported in the text). Detailed definitions of central constructs are provided in a supplementary glossary (see: 10.1007/s11920-020-01208-6). In the final section, we describe a working model and discuss limitations, open research questions, and clinical implications.

**Table 1:** List of most most relevant studies including information on study design, samples, sample sizes, paradigms, and most relevant findings with respect to the current topic

Author	Title	Year of publication	*N* (total)	Females (total)	*N* (HC)	Females (HC)	*N* (patients/ offenders)	Females (patients/ offenders)	Paradigm (s) and measures	Stimulus material	Main results
Achterberg M et al.	Control your anger! The neural basis of aggression regulation in response to negative social feedback.	2016	30	15	30	15	0	0	Modified SNAT; fMRI	Digital sham peer feedback coupled to profile picture	Higher aggression after negative feedback in the SNAT. Involvement of dlPFC in regulation of impulsive actions
Bertsch K et al.	Oxytocin and reduction of social threat hypersensitivity in women with borderline personality disorder.	2013	81	81	41	41	40 BPD	40	Facial emotion classification paradigm; fMRI	Static, validated emotional faces	In subjects with BPD, stronger amygdala activations were found in response to facial anger vs. happiness. Amygdala reactivity to anger was related to faster saccades into the eyes of angry faces.
Bertsch K et al.*	Neural correlates of emotional action control in anger-prone women with borderline personality disorder.	2018	58	58	28	28	30 BPD	30	Emotional AAT; fMRI	Static, validated emotional faces	BPD patients were faster in approaching than avoiding angry faces and showed reduced vlPFC and dlPFC activity as well as reduced dlPFC-amygdala coupling during emotional action control.
Bertsch K et al.*	Out of control? Acting out anger is associated with deficient prefrontal emotional action control in male patients with borderline personality disorder.	2019	40	0	25	0	15 BPD	0	Emotional AAT; fMRI	Static, validated emotional faces	BPD patients showed reduced vlPFC activations during emotional action control. Anger out was negatively related to vlPFC and dlPFC activation, but positively to amygdala activity in BPD patients.
Beyer et al.	Orbitofrontal cortex reactivity to angry facial expressions in a social interaction correlates with aggressive behavior.	2015	32	0	0	0	0	0	STAP; fMRI	Auditory punishment by opponent; video clips displaying angry or neutral faces	Medial OFC reactivity to angry faces correlated negatively with aggressive behavior. Individual variance in ACC activity correlated positively with aggressive behavior to angry expressions.
Buades-Rotger M et al.	Endogenous testosterone is associated with lower amygdala reactivity to angry faces and reduced aggressive behavior in healthy young women.	2016	39	39	39	39	0	0	STAP; fMRI	Auditory punishment by opponent; video clips displaying angry or neutral faces	Reduced BLA-OFC coupling was observed at presentation of angry vs. neutral facial expressions. BLA reactivity was positively related to aggression.
Buades-Rotger M et al.	Trait and state patterns of basolateral amygdala connectivity at rest are related to endogenous testosterone and aggression in healthy young women.	2019	39	39	39	39	0	0	STAP; resting-state fMRI	Auditory punishment by opponent; static images displaying angry or neutral faces	Stronger increase in vmPFC/medial OFC-amygdala resting-state connectivity after an aggressive encounter was related to reduce aggression.
Chester DS et al.	The interactive effect of social pain and executive functioning on aggression: an fMRI experiment.	2014	35	17	35	17	0	0	Stroop color-naming task, Cyberball paradigm, modified TAP; fMRI	Rejection or inclusion in a virtual ball-tossing game; auditory punishment by opponent	In individuals with low executive functioning increased dACC and anterior insula activation predicted increased aggression, while it predicted decreased aggression in participants with high executive functioning.
Chester DS et al.	Physical aggressiveness and gray matter deficits in ventromedial prefrontal cortex.	2017	138	91	138	91	0	0	sMRI	n.a.	Reduced volume and density of gray matter in the vmPFC were associated with physical aggression and increased likelihood of participation in real-world violence.
Chester DS et al.	Neural mechanisms of the rejection-aggression link.	2018	60	38	60	38	0	0	Cyberball paradigm; modified TAP; fMRI	Rejection or inclusion in a virtual ball-tossing game; auditory punishment by opponent	vlPFC recruitment during rejection was associated with increased striatal activity at retaliation, which was in turn associated with greater levels of retaliatory aggression. Functional connectivity between the VS and the right vlPFC during aggression reduced aggressive behavior and was impaired in dispositionally aggressive participants.
Chester DS et al.	Neural correlates of intertemporal choice in aggressive behavior.	2019	61	37	61	37	0	0	Modified TAP; fMRI	Auditory punishment by opponent	Selecting delayed but greater aggressive responses instead of immediate but lesser aggression was associated with greater vmPFC activity. Immediate but less severe aggressive responses were correlated with reduced connectivity between vmPFC and the fronto-parietal network (dlPFC, superior parietal lobule).
Choe DE et al.*	Maladaptive social information processing in childhood predicts young men’s atypical amygdala reactivity to threat.	2015	310; longitudinal assessments from ages 10 to 22	0	310	0	0	0	Laboratory task battery; social threat task; criminal arrest records; fMRI;	Illustrated vignettes of aggression directed against hypothetical self; static, validated emotional faces	Impaired social information processing at ages 10 and 11 predicted increased amygdala reactivity to fearful faces at age 20.
Coccaro EF et al.	Morphometric analysis of amygdala and hippocampus shape in impulsively aggressive and healthy control subjects.	2015	140	78	73	44	67 IED	34	sMRI	n.a.	Amygdala and hippocampus deformations in subjects with IED compared to healthy subjects were correlated with elevated aggression in IED.
Coccaro EF et al.	Frontolimbic morphometric abnormalities in intermittent explosive disorder and aggression.	2016	168	85	53	28	115 (57 IED, 58 other DSM-V diagnosis)	57	sMRI	n.a.	Smaller volumes in amygdala, OFC, vmPFC, ACC, insula, and uncus in subjects with IED compared to healthy subjects were correlated with elevated aggression in IED.
da Cunha-Bang S et al.*	Violent offenders respond to provocations with high amygdala and striatal reactivity.	2017	44	0	26	0	18 violent offenders	0	PSAP; fMRI	Provocation by opponent by withdrawal of monetary units	Violent offenders displayed more aggressive behavior and increased amygdala and striatal reactivity to provocations. Connectivity was reduced between amygdala and right superior prefrontal gyrus and between striatum and medial OFC. In the total group striatal and PFC reactivity to provocations was positively associated with trait anger and trait aggression.
da Cunha-Bang S et al.	Amygdala reactivity to fearful faces correlates positively with impulsive aggression.	2019	47	0	28	0	19 violent offenders	0	Facial emotion classification paradigm; fMRI	Static, validated emotional faces	Impulsive aggression was associated with amygdala reactivity in response to fearful but not angry faces.
Deveney et al.	Neural mechanisms of frustration in chronically irritable children.	2013	42 adolescents; aged 8–17 years	16	23	12	19 with severe mood dysregulation	4	Modified affective Posner Task; fMRI	Automated negative feedback despite correct performance	During the frustration condition, individuals in the severe mood dysregulation group displayed abnormally reduced activation in the left amygdala, bilateral striatum, parietal cortex, and posterior cingulate.
Farah T et al.	Alexithymia and reactive aggression: the role of the amygdala.	2018	156	0	156	0	0	0	sMRI	n.a.	Right amygdala volume positively correlated with alexithymia and reactive aggression but not proactive aggression.
FitzGerald TH et al.	Reward-related activity in ventral striatum is action contingent and modulated by behavioral relevance	2014	25	17	25	17	0	0	Monetary reward task modulated by sensory stimuli; fMRI	Emotionally neutral visual and auditory stimuli signaling offer value	Value of behaviorally relevant stimuli was correlated with activations of the VS as part of the brain’s reward network.
Gilam G et al.	Attenuating anger and aggression with neuromodulation of the vmPFC: a simultaneous tDCS-fMRI study.	2018	25	15	25	15	0	0	Modified UG; TAP; anodal transcranial direct current stimulation (tDCS); fMRI	Written provocations coupled with unfair monetary offers; auditory punishment by opponent	Transcranial stimulation of the vmPFC promoted an increase in vmPFC activity while processing unfair offers, increased the acceptance of such offers and was associated with a lower increase in self-reported anger. Subsequent aggressive behavior was also attenuated after stimulation.
Herpertz SC et al.*	Brain mechanisms underlying reactive aggression in borderline personality disorder-sex matters.	2017	112	63	56	30	56 BPD	33	Script-driven imagery task of rejection and aggression; fMRI	Audiotaped stories on interpersonal rejection of hypothetical self, followed by physical aggression directed against others	Male BPD patients showed stronger dlPFC and lateral OFC activation than healthy men and female BPD patients while imagining acting out aggressively. Prefrontal-amygdala connectivity was negatively associated with trait anger in male BPD.
Hofhansel et al.	Morphology of the criminal brain: gray matter reductions are linked to antisocial behavior in offenders	2020	54	0	27	0	27 criminal offenders	0	sMRI	n.a.	Antisocial behavior was negatively correlated with gray matter volume in the right superior frontal and left inferior parietal regions in criminal offenders. Gray matter volume in the right middle and superior temporal gyrus was negatively correlated with both reactive aggression and antisocial behavior.
Klimecki OM et al.	Distinct brain areas involved in anger versus punishment during social interactions.	2018	25	0	25	0	0	0	IG (unfair economic game); fMRI	Unfair economic offers coupled with written provocations, fair economic offers coupled with friendly messages	Self-reported anger was positively related to activations in regions of the ToM network (i.e., temporal areas and precuneus) and the amygdala in response to an unfair co-player’s face. Activations in the dlPFC at provocation of anger predicted the inhibition of subsequent punishment.
Mancke F et al.	Amygdala structure and aggressiveness in borderline personality disorder	2018	109	65	51	28	58 BPD	37	sMRI	n.a.	Aggression in anger-prone men with BPD tended to positively correlate with right amygdala volume and displayed a correlation with shape deformations in the left superficial and laterobasal amygdala.
McCloskey MS et al.	Amygdala hyperactivation to angry faces in intermittent explosive disorder	2016	40	16	20	8	20 IED	8	Facial emotion classification paradigm; fMRI	Static, validated emotional faces	IED had increased amygdala responses to angry vs. neutral facial expressions. Independent of group, amygdala activation to angry faces was associated with prior aggressive acts as assessed by the Life History of Aggression Scale.
Ueltzhöffer K et al.	Whole-brain functional connectivity during script-driven aggression in borderline personality disorder	2019	63	63	30	30	33 BPD	33	Script-driven imagerytask; fMRI	Audiotaped stories on interpersonal rejection of hypothetical self, followed by physical aggression directed against others	Findings in individuals with BPD suggested an increased interaction of prefrontal cognitive control processes with thalamo-cortico-striatal action-selection processes.
Volman I et al.	Anterior prefrontal cortex inhibition impairs control over social emotional actions	2011	24	0	24	0	0	0	Emotional AAT; continuous theta burst stimulation (cTBS) inhibition of the left anterior PFC; fMRI	Static, validated emotional faces	Participants committed more errors in affect-incongruent trials when the vlPFC was inhibited by cTBS. Task-related perfusion was decreased in bilateral vlPFC and posterior parietal cortex and increased in amygdala and left fusiform face area.
Volman I et al.	Testosterone modulates altered prefrontal control of emotional actions in psychopathic offenders [1–3]	2016	34	0	19	0	15 violent offenders	0	Emotional AAT; fMRI	Static, validated emotional faces	Violent offenders exhibited less vlPFC activity and less vlPFC–amygdala connectivity during trials requiring emotional control.
White SF et al.*	Disrupted expected value and prediction error signaling in youths with disruptive behavior disorders during a passive avoidance task	2013	38 adolescents aged 10–18 years	11	18	8	20 DBD	3	Passive Avoidance Task; fMRI	Emotionally neutral visual stimuli signaling value	Adolescents with DBD showed reduced sensitivity to expected value information within the vmPFC when choosing objects and within the anterior insular cortex when refusing objects. These participants also exhibited reduced modulation within the caudate by prediction error of responses to reward but increased modulation within the caudate by prediction error of responses to punishment.
White SF et al.	Punishing unfairness: rewarding or the organization of a reactively aggressive response?	2014	21	9	21	9	0	0	Modified UG; fMRI	Fair/unfair economic offers	Higher punishment was associated with increasing activity within caudate, dorsomedial frontal cortex, anterior insular cortex, and PAG as well as decreasing activations in vmPFC and posterior cingulate cortex.
White SF et al.*	Neural correlates of the propensity for retaliatory behavior in youths with disruptive behavior disorders.	2016	56 adolescents aged 10–18 years	23	26	12	30 DBD	11	Modified UG; fMRI	Fair/unfair economic offers	Youth with DBD exhibited reduced vmPFC responsivity and reduced amygdala-vmPFC coupling during high provocation. These effects were related to patients’ higher retaliatory behavior and parent reported reactive aggression.
Yang Y et al.	Neural correlates of proactive and reactive aggression in adolescent twins.	2017	106 adolescents aged 14 years	52	106	52	0	0	sMRI	n.a.	Increased volumes in the caudate nuclei, putamen, and the nucleus accumbens were associated with both proactive and reactive aggression. Additionally, reduced volumes in the middle frontal cortex and the anterior cingulate cortex as well as increased volumes in the OFC and the insula were associated with higher levels of total aggression.

n.a. Not applicable

* Of importance

*AATSS* anger articulated thoughts during simulated situations paradigm, *AAT* approach avoidance task, *ACC* anterior cingulate cortex, *BLA* basolateral amygdala, *BPAQ* Buss-Perry Aggression Questionnaire, *BPD* borderline personality disorder, *dACC* dorsal anterior cingulate cortex, *DBD* disruptive behavior disorder, *dlPFC* dorsolateral prefrontal cortex, *fMRI* functional magnetic resonance imaging, *G/NG-T* go/no-go task, *IED* intermittent explosive disorder, *IG* inequality game, *OFC* orbitofrontal cortex, *PAG* periaqueductal gray, *PSAP* Point Subtraction Aggression Paradigm, *sMRI* structural magnetic resonance imaging, *SNAT* Social Network Aggression Task, *SSRT* stop-signal-reaction-time task, *STAP* Social Threat Aggression Paradigm, *TAP* Taylor Aggression Paradigm, *UG* ultimatum game, *vlPFC* ventrolateral prefrontal cortex, *VS* ventral striatum, *vmPFC* ventromedial prefrontal cortex

## Threat Sensitivity

Threat elicits fear of harm followed by avoidance tendencies, but can also provoke reactive aggression [8–10]. In the animal model so-called defensive, fear-driven, hyperarousal-associated aggression has been extensively studied and occurs in situations challenging survival or defending limited supply in rivalry conditions [11] and is related to increased activity in the medial amygdala [12]. Since in rodents, this activity was positively related to aggression and associated automatic stress responses in a post-weaning social isolation model, this finding may point out a possible brain mechanism for human threat-related aggression in clinical groups with a history of childhood maltreatment [13].

In animals and humans, the likelihood of aggression in response to social threat depends on both situational and trait factors. Regarding situational factors, aggressive attack occurs in reaction to intense, unescapable social threats [10]. While Blanchard claims a distinct “fear of harm” phenotype of human aggression which is associated with the emotion of fear, others suggest that fear-related escape behavior is likely to turn into anger-related approach and, thus, aggressive behavior in the face of inescapable threat [8]. Whenever circumstances permit, individuals tend to calculate the value of response and gauge consequences of attack or escape in response to social threat with trait factors such as approach-avoidance tendencies moderating decision-making. While aggressive approach may be adaptive in the context of inescapable threat, individuals with high fear reactivity or strong avoidance tendencies are more likely to seek escape than to attack [14]. Particularly, clinical groups, most typically individuals with borderline personality disorder (BPD), tend to respond to even slight interpersonal challenge as though it was an inescapable threat [15], direct more attention to social threat cues [16], and show a hostile attribution bias [17], altogether increasing the likelihood for approach rather than avoidance behavior to potentially threatening cues. Importantly, in humans, situation and trait factors indissolubly interact: a threat cue may be perceived or interpreted as threatening by some but not by others. Particularly, clinical populations with a history of early maltreatment, as frequently found in BPD or antisocial personality disorder (ASPD), tend to be hypersensitive to social threat on the background of previous experiences of overwhelming threat that may trigger fight responses on the behavioral, neuronal, autonomic, and hormonal level [18]. Furthermore, fear and avoidance may turn into anger and approach behavior or individuals may rapidly oscillate between both emotions and action tendencies within situations they experience as threatening [8].

Core structures of the brain’s threat circuitry subsume the amygdala, hypothalamus, and periaqueductal gray (PAG). The amygdala plays a key role in the processing of arousal and emotions including fear and anger. It facilitates behavioral, hormonal, and autonomic “survival” actions to imminent threat via the hypothalamus and PAG, while in the case of more distant or possibly escapable threat, it is likely to interact with prefrontal and striatal regions. In such situations, interactions of the amygdala with the ventromedial prefrontal cortex (vmPFC; involved in gathering action values and contingency information), the anterior insula (as part of the brain’s salience network), and the anterior cingulate cortex (ACC; related to motor response selection), allow for a more thorough evaluation of the current threat situation and a cognitively controlled emotional and behavioral response [19]. Particularly, the vmPFC modulates the threat response, so that it is turned down when realizing that something—“at second sight”—does not actually indicate danger, or is amplified in case of previously learnt contingencies are activated, as it happens in early stress related disorders [20]. Hence, childhood maltreatment, by affecting the development of the threat response system and its linkage with prefrontal areas, reprograms response to real or perceived threat cues in later life [20].

An association between reactive aggression and the amygdala is suggested by structural imaging data showing a positive correlation between reactive aggression and right amygdala volume in a large community sample [21]. Furthermore, smaller amygdala volume [22] and amygdala surface shape deformations [23] were related to elevated aggression in subjects with intermittent explosive disorder (IED), a disorder defined by reactive aggression. By contrast, larger volume and shape deformations of the amygdala correlated with aggression in anger-prone men with BPD [24].

In experimental studies, the threat circuitry is typically stimulated using pictures of fearful or angry faces. By communicating hostility, angry faces are used as a direct proxy for social threat [25], while fearful faces evoke attentional monitoring of a threatening environment and thus ambiguous threat [26]. In a large study in 10–12-year-old boys, the tendency to respond to ambiguous social scenes with hostility attribution and reactive aggression predicted participants’ amygdala response to fearful faces in an emotional face matching task in young adulthood [27•]. This suggests an association between maladaptive social threat detection and aggressive responding [27•] and is consistent to a positive relationship between basolateral amygdala responses to angry faces and aggressive responses in healthy women undergoing the Social Threat Aggression Paradigm (STAP; [28]). In the STAP, a competitive reaction time task similar to the Taylor Aggression Paradigm (TAP), which however includes the presentation of brief video clips displaying an angry or neutral looking opponent just before the participant’s punishment selection, amygdala activity was positively associated with aggression (i.e., the administered loudness of an aversive auditory punishment) in trials where the opponent showed angry but not neutral facial emotions [28]. Notably, in an inequality game (IG), self-reported anger was positively associated with amygdala activity when viewing the face of an unfair other who did not look angry but represented threat to the participant’s self-worth by giving derogatory social feedback (*N* = 25 healthy volunteers; [29]). In this competitive economic game, anger is induced by an unfair other player who gives derogatory feedback messages as opposed to a fair other player who behaves cooperatively.

Enhanced amygdala activity to angry faces and positive correlations between amygdala response and aggressive behavior in everyday life has been found in IED [30]. In BPD, amygdala reactivity was related to fast threat responses, i.e., faster saccades to the eyes of angry faces [16], which were in turn positively correlated with trait aggression [31]. A positive association was also found between trait aggression and amygdala reactivity to fearful faces in violent offenders [32].

Experimental provocation paradigms, such as the TAP, STAP, the Point Subtraction Aggression Paradigm (PSAP), or economic games (e.g., IG or ultimatum game (UG)), all comprise some forms of physical or monetary punishment or unfair treatment by another person within an interpersonal competition. Increment of activations found in the amygdala, hypothalamus, and PAG in a number of studies using these game paradigms might suggest that the brain’s threat response system is involved in provocation [19]. In line with this, in healthy individuals, PAG activation increased with higher levels of punishment in an UG, which creates provocation in form of unfair offers (*N* = 21 healthy volunteers; [33]). Enhanced amygdala activity to provocation has also been reported in highly aggressive groups. For instance, in adolescents with disruptive behavior disorders (DBD), amygdala and PAG responses to provocation correlated positively with retaliatory behavior in the UG [34•]. Furthermore, violent offenders showed increased amygdala response to provocation in the PSAP, where aggression is provoked by a fictitious opponent stealing points from the participant’s account. Amygdala activity correlated positively with task-induced aggressive behavior that is stealing points without gaining anything for oneself [35]. Notably, not all studies have found amygdala activations in response to provocation [36]. Discrepant findings may simply represent type II error or be attributable to task-related differences in amygdala habituation or cognitive demands.

## Frustrative Non-reward

Frustrative non-reward is a further trigger for anger and reactive aggression, subsuming reactions elicited by prevented or withdrawn rewards. As such, frustrative non-reward can be understood as an expectancy violation that is the outcome of a negative prediction error [37]. The core region mediating frustrative non-reward is the ventral striatum (VS), which acts in conjunction with the vmPFC, insula, dorsal ACC, and inferior frontal gyrus (IFG) [38]. The VS, as part of the brain’s reward network, is involved in learning about the predictive reward and reinforcement contingencies of (social) signals (*N* = 25 healthy volunteers; [39]).

Aggression in response to frustration typically occurs when one’s desired goal is blocked [40]. It has been suggested that retaliatory behavior, accompanied by feelings of revenge, may compensate for frustrative non-reward by enhancing VS activity (“sweetness of revenge”). For instance, violent offenders’ enhanced striatal activity was found to positively correlate with trait aggression and anger in the PSAP [35]. Furthermore, higher VS activity was reported in the decision phase of a competitive task similar to the TAP in which the loser was punished by an aversive noise and the intensity of the blast was determined by the opponent [41]. VS activity predicted the degree of retaliatory behavior in the game and was associated with the participant’s life history of violent behavior [41]. Activations in competitive tasks often involve not only the ventral but also the dorsal striatum (caudate nucleus), which mediates the selection and preparation of motor responses (e.g., retaliatory punishment). In an UG, increasing activity in the dorsal striatum was related to higher punishments of frustrative offers in healthy volunteers (*N* = 21 healty volunteers; [33]).

Furthermore, brain imaging data suggests that reactive aggression resulting from frustration and provocation is modulated by control areas of the vmPFC and lateral prefrontal cortex (PFC). The vmPFC mediates processing reward vs. punishment for regulatory purposes in conflicting decision-making, with an impact on automatic and instrumental action tendencies [19]. Correspondingly, enhanced acceptance of unfair offers and dampened anger response in the UG were found after transcranial stimulation of the vmPFC (*N* = 25 healthy volunteers; [42]), and physical aggression correlated with smaller vmPFC [43] but larger striatal [44] volume in healthy participants. Additionally, retaliatory aggression in response to social exclusion was associated with VS activity and connectivity to the ventrolateral prefrontal cortex (vlPFC) [45]. Connectivity was reduced in aggressive participants, consistent with the vlPFC’s role in emotional action regulation [45].

An involvement of a dysregulated threat network along with abnormal activities in reward structures in response to frustration is suggested by some clinical studies. In youth with DBD, frustrative non-reward induced by highly unfair offers in an UG was associated with enhanced amygdala and PAG activity and, thus, a hyperactive threat circuit, as well as reduced vmPFC-amygdala connectivity. Interestingly, this neuronal pattern was correlated with task-induced retaliatory behavior and parent-reported reactive aggression [34•]. Youths with DBD also showed reduced use of expected value information within the vmPFC in a decision-making task where reinforcement was probabilistic that is over the course of the experiment, two cues were followed by feedback of gain and two other cues by feedback of loss [46•]. In this study, youths with DBD also showed enhanced responsiveness to frustration, namely, negative prediction errors (punishment was greater than expected) during feedback which was processed in the caudate [46•]. However, in a study of youth with disruptive mood dysregulation disorder (DMDD), frustrative non-reward was not associated with increased amygdala activity but, on the contrary, with decreased amygdala activity in response to negative social feedback in a cued-attention task performed under frustrating and non-frustrating conditions [47].

## Cognitive Control

Social threat, provocation, and frustration trigger negative feelings, such as anger and a behavioral impulse to an aggressive approach. However, oftentimes, individuals do not act out aggressively in such situations. Here, cognitive control, a set of higher-order regulatory functions primarily mediated by the prefrontal cortex [48] that support goal-directed behavior by modulating other cognitive and emotional processes, comes into play [49]. So far, empirical evidence from both neuroscience and clinical studies particularly underlines deficits in two cognitive control functions, namely, inhibitory control and emotion regulation [50, 51]. While inhibitory control or behavior control implies the inhibition of fast, premature, uncontrolled behavioral responses to pursue goal-directed behavior [48], emotion regulation or emotion control is defined as the ability to downregulate negative emotions [52]. In aggressive situations, dysregulated emotions aggravate poor behavior control. Thus, both, behavior and emotion control, are mediated by a broad fronto-parieto-insular network of brain regions implicated in monitoring, selecting, modulating, and evaluating behavioral/emotional responses, namely the dorsomedial prefrontal cortex (dmPFC)/dorsal anterior cingulate cortex (dACC)/pre-supplementary motor area (preSMA), bilateral anterior insula reaching into the IFG/vlPFC, and inferior parietal lobules (IPL).

Very recently, negative associations between gray matter volume in regions belonging to this cognitive control network (i.e., right super frontal gyrus, right middle and superior temporal regions, and left inferior parietal lobe) and antisocial behavior—and for the right temporal lobe—reactive aggression have been reported in male criminal offenders [53]. Furthermore, stronger activations in parts of this system have been found in response to high vs. low levels of provocation or videos showing an angry vs. neutral looking opponent with most consistent activations in the dmPFC and dACC across functional neuroimaging studies [36]. In two studies, healthy participants showed elevated activations in dmPFC/dACC, IFG, and superior temporal gyrus in response to angry vs. neutral looking opponents in the STAP [28, 54]. These regions have been implicated in monitoring conflicts between desired and actual actions (dmPFC), response monitoring and error processing (dACC together with anterior insula), and the selection of context-appropriate and inhibition of context-inappropriate response (IFG/vlPFC together with inferior parietal lobules [55]). In close interaction with the inferior parietal lobules (IPL), the IFG is involved in modulating excitatory circuits of preSMA and subthalamic nucleus, thus leading to enhanced inhibition from the subthalamic nucleus to the motor cortex [56] and to subcortical regions implicated in automatic, stereotyped emotional action impulses, i.e., the amygdala, VS, hypothalamus, and PAG. Correspondingly, choosing an aggressive response for an opponent in the STAP involves a set of cognitive control processes and response selection. High levels of social threat raised by the opponent’s angry expression and high provocation may intertwine in increasing the demand of inhibiting the urge to act out impulsively during the STAP in normal volunteers [28].

When having to inhibit fast amygdala-driven emotional action tendencies in approach-avoidance tasks, aggression-prone patients with BPD [57•, 58•] and violent offenders [59] were found to show lower recruitment of the vlPFC and dlPFC, which has been related to the tendency to act out anger [58•]. In these tasks, participants are instructed to either approach or avoid an angry or happy facial expression by pulling or pushing a joystick. Inhibiting fast tendencies to approach happy and avoid angry faces in order to perform the opposite behavior has been consistently related to an increase in lateral PFC activations in healthy volunteers [60]. The inhibition of vlPFC activity with transcranial magnetic stimulation resulted in worse performance and increased amygdala activity in healthy male participants. This supports the vlPFC’s direct involvement in emotional action control and response selection by integrating and coordinating different cognitive processes (*N* = 24 healthy volunteers; [61]), while the dlPFC has been associated with keeping control strategies and goals in mind and directing attention to relevant perceptual inputs, a continuous updating and manipulation of stimuli in working memory, and emotion regulation [62, 63]. In another study, in which healthy participants adjusted the intensity of an aversive noise blast after receiving negative, positive, or neutral feedback from an alleged co-player, elevated right dlPFC activation to negative feedback was related to lower levels of retaliation [64]. Furthermore, male patients with BPD showed stronger dlPFC and lateral orbitofrontal cortex (OFC) activations compared to controls while imagining acting out aggressively [65•]. Since the prefrontal-amygdala connectivity of men with BPD was negatively associated with trait anger [65•], this suggests poor top-down adjustment despite efforts of control. Here, participants were instructed to vividly image brief stories describing neutral situations, anger-inducing interpersonal rejections, and subsequent acts of physical aggression against the provocateur. In the same study, female patients with BPD compared to healthy controls showed stronger increase in connectivity within a large brain network that suggested increased interaction of prefrontal cognitive control processes with thalamo-cortico-striatal action-selection processes while processing aggressive actions [66]. A stronger increase in connectivity between regions of the cognitive control network and regions of the motor system might be interpreted as the patients’ attempts to control aggressive action impulses. These results may thus reflect a tendency to try and compensate ineffective emotion regulation strategies by directly suppressing aggressive action impulses [66].

There are also reports of reduced functional coupling between the amygdalae and the vmPFC/medial OFC (mOFC) in response to angry faces in healthy individuals [28], suggesting differences in the coupling between threat system (amygdalae) and contingency processing (vmPFC/mOFC). As mentioned above, individuals are required to choose a more or less aggressive response for their opponent while facing this person’s threatening expression. In healthy individuals, a stronger increase in vmPFC/mOFC-amygdala resting-state connectivity after the STAP was related to reduced aggression [67]. Furthermore, selecting delayed but greater aggressive responses (i.e., louder noise blast) instead of immediate but lesser aggression (i.e., softer noise blast) in a modified TAP was associated with greater vmPFC activity and an elevated connectivity with the fronto-parietal network in healthy subjects [68], probably reflecting higher cognitive load when thinking about contingencies [19]. These reports are accompanied by reports of reduced gray matter volumes in the vmPFC/OFC, ACC, amygdala, insula, and uncus in individuals with IED which were correlated with measures of aggressive behavior [22] and reports of severe deficits in behavior and emotion control in individuals with prefrontal lesions [69, 70].

As noted above, healthy participants showed stronger dACC activations in trials with high vs. low levels of provocation across studies [36] in line with this region’s involvement in emotion regulation, response monitoring, and error processing. Classically, the so-called Go/No-Go tasks, which require a response to certain frequency and a response inhibition to infrequent stimuli, have been used to investigate behavior control. During such a task, deficient error processing, in terms of smaller event-related EEG potentials generated in the dACC, has been reported in highly aggressive individuals [71], and strong associations between trait anger and deficits in inhibitory control and automatic error processing were found in forensic patients during hostile conditions of an affective Go/No-Go task [72]. Furthermore, the interaction of behavior control assessed in a color-naming Stroop task and dACC/anterior insula response to social rejection in a Cyberball game predicted reactive aggression in healthy participants [73]: While individuals with low behavior control showed a positive association between insula/dACC activations and aggression, this association was negative in individuals with high behavior control. The results hence indicate an interference of negative emotional states triggered by provocation, rejection, and other negative interpersonal experiences with cognitive functions in individuals with low inhibitory control, which may lead to impulsive aggressive behavior. This is in line with evidence from the IMAGEN consortium on neural correlates of behavioral inhibition in 1709 participants according to which the lateral OFC is particularly involved in the processing of stop signals that inhibit actions, the dlPFC in attentional processes influencing task performance, and the anterior insula and ACC in emotional processes related to failure [74].

Thus, reactive aggression is likely to occur in individuals in whom poor inhibitory control co-occurs with deficient emotion regulation. Refraining from aggressive actions when socially threatened, provoked, or frustrated requires the capacity to downregulate negative emotions and inhibit the urge to act outs aggressively. The stronger the elicited emotions, the higher the need for cognitive control. Notably, cognitive control is not always reliable and/or tends to become fatigued with frequent use, rendering some individuals prone to aggressive acts. Self-report data confirm deficits in inhibitory control [75–77] and in emotion regulation [78–80] in highly aggressive individuals who also seem to benefit less from explicit instructions to down-regulate negative emotions with the so-called adaptive strategies, such as reappraisal or distancing [81]. While for patients with BPD, in whom aggression has been shown to only occur in highly emotional contexts, emotion dysregulation is of particular importance for reactive aggression, deficient behavior, and emotion control might be equally relevant in ASPD and IED [51]. On a neural level, social threat, provocation, or frustration may challenge the core fronto-parieto-insular network implicated in behavior and emotion control. In these situations, fast action tendencies of approach or avoidance driven by subcortical circuits dominate rendering vulnerable subjects prone to act out on the urge to aggress. Results from functional and structural and lesion studies suggest a particular relevance of the PFC in pursuing goal-driven behavior maybe by sending bias signals to other brain areas that favor relevant sensory input, memories, and motor output [48].

## Working Model and Open Research Questions

**Fig. 1:**
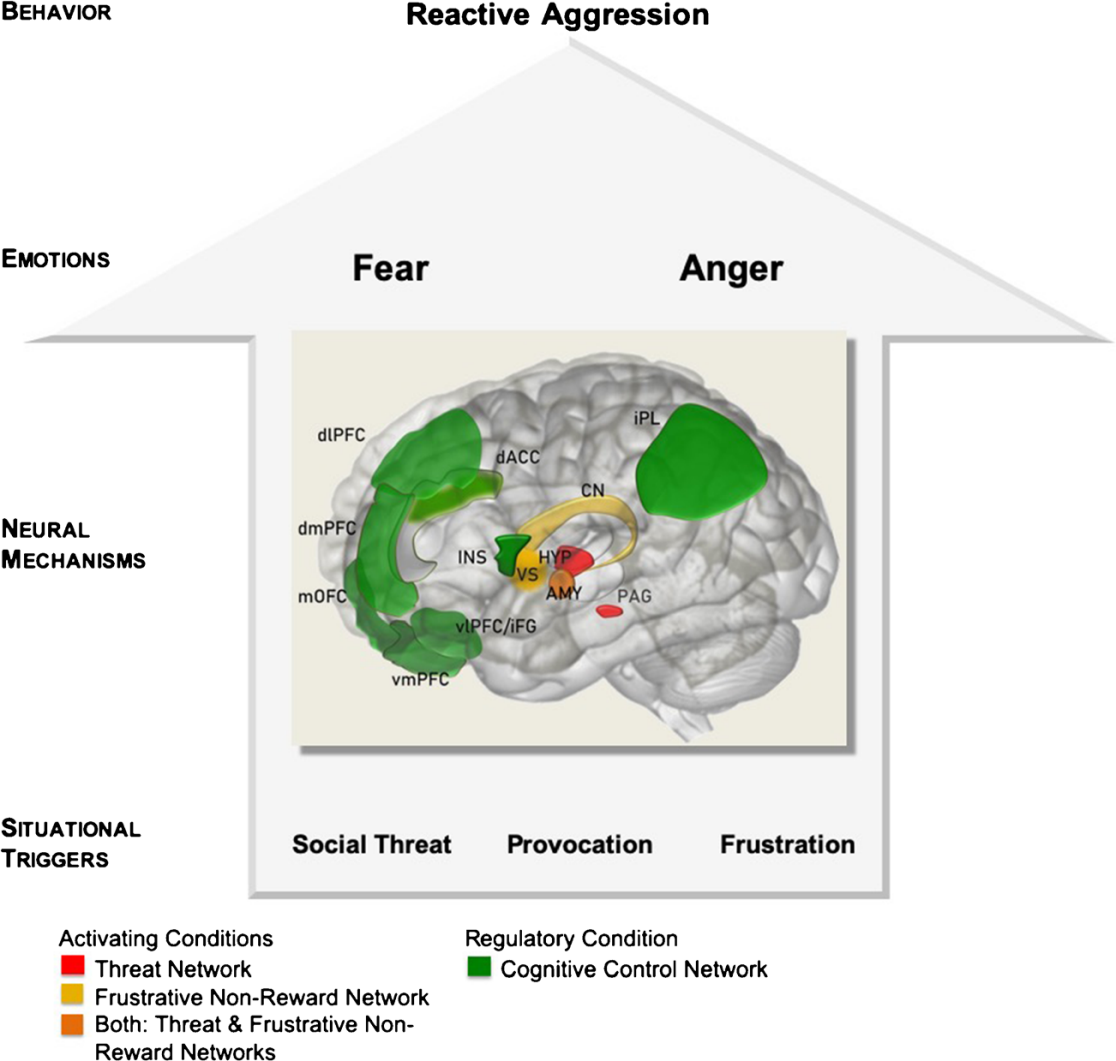
Working model of reactive aggression representing major brain regions underlying threat sensitivity and frustrative non-reward as activating conditions and cognitive control as regulating condition of reactive aggression. AMY, amygdala; CN, caudate nucleus; HYP, hypothalamus; dACC, dorsal anterior cingulate cortex; dlPFC, dorsolateral prefrontal cortex; dmPFC, dorsomedial prefrontal cortex; IFG, inferior frontal gyrus; INS, insula; IPL, inferior parietal lobules; mOFC, medial orbitofrontal cortex; PAG, periaqueductal gray; vlPFC, ventrolateral prefrontal cortex; vmPFC, ventromedial prefrontal cortex; VS, ventral striatum

We systematically summarized and structured the recent literature in three sections on threat sensitivity and frustrative non-reward as activating conditions and cognitive control subsuming regulatory mechanisms to search for transdiagnostic biobehavioral mechanisms. Based on these sections, we will formulate a working model for future research designs trying to capture the multidimensional nature of reactive aggression and hypothesizing about pathways from the typical situational triggers social threat, provocation, and frustration, via the three brain mechanisms, to behavior (Fig. 1). First, though, we would like to acknowledge some important limitations: (i) data on neural correlates of induced reactive aggression, particularly in highly aggressive or clinical groups, are still limited, which may be strongly related to (ii) difficulties in reliably inducing reactive aggression in the MRI environment. (iii) Existing studies revealed an array of activated brain regions which have been interpreted in various ways based on a priori hypotheses, the particular paradigm used, or the specific functional deficits of the included clinical group. Different task demands which may also explain various activation patterns have not fully been addressed in the current review. (iv) Although we focused on studies in which aggression is explicitly measured, the heterogeneity and variation in the symptomatology of clinical phenotypes need to be taken into account, in addition to the correlational nature of the presented brain imaging results. Together with well-known limitations of MRI in terms of temporal and spatial resolution, small samples of patients with heterogenous clinical disorders may tremendously limit the interpretability of available data. (v) Social threat, provocation, and frustration share important aspects, since all three elicit negative emotions and they can interact with one another. (vi) The selected conditions are not exclusive and many more processes may be involved in the activation and regulation of aggressive reactions, such as decision-making, theory-of-mind, or empathy. (vii) The current review does not address developmental aspects, or genetic and epigenetic mechanisms of reactive aggression [82–84]. It neither considers effects of sex hormones nor of vasopressin and oxytocin, although first promising effects on social threat, provocation, and frustration processing have been reported [16, 85–88]. For these reasons, the following working model should be regarded as a hypothesis to stimulate further research.

The brain’s threat network comprises the amygdala, PAG, and hypothalamus. An individual’s threshold for detecting social threats may be lowered by attentional threat biases or hostile attributions associated with faster, elevated, and/or prolonged responses of the threat circuity. Depending on individual differences and the proximity of the threat, a fronto-parieto-insular cognitive control network, in particular brain regions supporting emotion regulation and inhibitory control, may exert modulatory effects. Since economic game tasks that have been designed to provoke anger and retaliatory aggression were found to activate the threat circuit, provocation might be regarded as a specific form of social threat. In individuals with a predisposed sensitivity to provocation and poor (lateral) prefrontal emotion regulation capacities, as often found in clinical groups, provocation elicits strong feelings of anger, which in the case of deficient inferior fronto-parietal inhibitory control escalates into aggressive reactions, even in response to minor provocations [89]. Furthermore, social threat processing can interact with the evaluation of value and contingency (vmPFC), i.e., aggressive responses are less likely if the situational context, the aggressive action itself, and its consequences are not expected to provide reward. Furthermore, abnormal threat processing can interact with frustrative non-reward, as has been shown in irritable youth [90]. More knowledge on the rewarding nature of aggressive outbursts is needed. This is of particular relevance with regard to frustration, another prominent trigger for aggression. Although it remains difficult to distinguish its psychological and neurobiological effects from those of provocation, e.g., because both provoke a tit-for-tat-like retaliatory aggression. Other than povocation, frustration is closely associated with prediction errors and the brain’s reward system, with higher VS activity correlating with or predicting the degree of realiatory behavior particularly in individuals with low frustration tolerance [35, 41]. Retaliatory aggression following frustration may involve inadequate processing of response costs and abnormalities in reward and threat processing associated with dysfunctions in the VS [41] and the threat system [47], and reduced vmPFC-amygdala connectivity [34•] as seen when highly reactively aggressive clinical groups punish unfair opponents. Furthermore, reactive aggression following frustrative non-reward is modulated by regions associated with evaluation, and particularly theory-of-mind processes and thus the awareness of one’s own and other people’s intentions. This is in line with the social information processing theory of aggression [91], according to which frustration only results in aggressive retaliation when the frustrated individual attributes hostile intent to the opponent. Furthermore, decreased empathy, i.e., reduced sharing of other people’s negative emotions, and reduced compassion were reported in aggressive offenders [92]. Overlap also exists between frustrative non-reward and acute threat which, in the Research Domain Criteria (RDoC) matrix [93] both belong to the negative valence system and have been related to the same paradigms with blocking of an expected access to resources (e.g., competitive games, ostracism, or unfair treatment) as these may induce both frustration and threat. Evidence for a difference between these systems comes from studies that found competitive processes between regions responding to threat of shock and those engaged in decision-making about a monetary reward [94]. In irritable youths, threat hypersensitivity and frustrative non-reward are thought to interact [90].

As noted above, the current literature leaves open several important research questions that should be addressed in future studies. First, most of the currently used paradigms are not designed in a way to disentangle distinct motivations for aggressive behavior as proposed by the traditional differentiation between threat, provocation, and frustration [3]. We do not know whether hyperactivation of the basic threat circuit indeed reflects a specific threat response or rather a socially challenging and emotionally salient event in general [95] that is processed in the amygdala and affects physiology and behavior via downstream activations of hypothalamus and PAG [96]. This challenges the question whether provocation which has been shown to activate the threat circuit actually reflects a complex form of social threat to humans or just like social threat acts as a salient event. Thus, provocation, e.g., in the shape of unfair offers, may be perceived as harmful or as a salient event pointing to expectancy violation which results in frustration. To answer this question, more complex paradigms have to be developed in which the adaptiveness of social decision strategies can be quantified and used for computational modeling. Thereby, paradigms could be developed that allow a differentiation between threat and provocation, e.g., by varying the intentions of a co-player [97]. Second, it is also almost impossible to disentangle brain circuits related to frustrative non-reward from those related to provocation in commonly used economic game paradigms, since they measure retaliation in response to unfair and thus provocative offers that frustrate the participants’ reward expectancies. Economic game paradigms are the most frequently selected tasks to investigate frustration. While in these tasks, frustration is usually provided by human opponents, we do not know how aggression is related to non-social frustration (e.g., in case the PC does not work appropriately). Furthermore, both provocation and frustration elicit anger, which, however, may either result in explosive outbursts or in retaliatory behavior. Notably, the latter can be of impulsive or premediated, instrumental nature: While impulsive retaliation is likely associated with inadequate processing of response costs, premediated retaliation is rather an instrumental, intentionally selected response, congruent with reinforcement expectancies [3]. Consequently, future paradigms need to challenge impulsive as opposed to premediated retaliatory aggression within one study design. Third, we do not know yet whether threat-related aggression is directly provoked by the emotion of fear as suggested by the animal model of defensive aggression or implicates a move from avoiding toward approaching threat that is inseparably linked to a switch from fear to anger. fMRI tasks of social threat that combine the measurement of aggression level with queries on accompanying emotions of anger and fear may contribute to answer this issue. Forth, based on animal results, it might be interesting to study whether reactive aggression is associated with aberrant perceptions of the proximity of a threat in future studies. To our knowledge, this has not been addressed in humans, yet, despite the availability of MRI paradigms which allow the manipulation of threat proximity [98]. Fifth, it remains difficult to disentangle the involvement of specific cognitive control functions in the regulation of reactive aggression based on the current literature. Tight interactions between behavior and emotion control have been described and first results in healthy participants suggest that problems in emotion regulation might be particularly problematic when individuals with low inhibitory control are facing threats, provocations, or frustrations [73]. Finally and more generally, further effort is needed regarding the measurement of reactive aggression. Aside from valid reports about real-life aggression, it is necessary to develop new, ecologically more valid experimental paradigms that systematically manipulate social context information and include behavioral options other than aggression. Moreover, technological advances in high-resolution fMRI and machine learning should be utilized in order to better tackle the complex neural mechanisms of reactive aggression and to further disentangle specific psychological and neural mechanisms of reactive aggression. This would provide the chance to develop mechanism-based treatments that specifically address the individual patient’s needs, given that so far, treatment approaches have only shown small effect sizes [99, 100]. A detailed diagnostic assessment including experimental measures of threat sensitivity, frustrative non-reward, and cognitive control could be helpful for implementing modularized psychotherapy and targeted pharmacotherapy.

## Conclusions

For clinical samples with high aggression and prominent threat sensitivity, improving prefrontal-amygdala inhibition in response to alleged threats, provocations, and frustrations might be a promising target for interventions. Correspondingly, activations in social threat and emotion control circuits have already been shown to be susceptible to psychotherapy [101], brain stimulation [42, 61], and neurofeedback [102]. However, more specific interventions are needed to target the specific mechanisms underlying reactive aggression in this population (Herpertz et al., under review).

## Supplementary Information

ESM 1 (DOCX 42 kb)
