# Supplementary material for: Understanding Brain Mechanisms of Reactive Aggression
Source: Curr Psychiatry Rep. 2020 Nov 12;22(12):81. doi: 10.1007/s11920-020-01208-6 (PMC7661405; doi:10.1007/s11920-020-01208-6)
Supplement: Supplementary file 1 — (DOCX 42 kb) [file 11920_2020_1208_MOESM1_ESM.docx]

**Glossary**

*Forms of aggression*

*Aggression:* any behavior that is carried out with the intent to cause harm to another individual, who is motivated to avoid this harm (1).

*Explosive aggression:* impulsive reactions to (minor) provocations that are regarded as inappropriate and may cause damage to other individuals or valuable objects. Explosive aggression is a key feature of individuals with intermittent explosive disorder (2).

*Defensive aggression:* a construct which has been deduced from animal data; mostly non-pathological, defensive attack behavior that becomes more likely with increasing proximity, imminence, severity, and inescapability of a real or perceived threat (3). Although being originally fear-driven, it may also go along with anger in humans (4).

*Proactive, premeditated, instrumental aggression*: a planned, unemotional, goal-directed behavior that causes harm to others (1). Instrumental aggression is cold-blooded and mostly found in individuals with high levels of psychopathy.

*Reactive, impulsive aggression:* a non-goal driven, retaliatory, or defensive behavior in response to a perceived threat, frustration, or provocation (1). Reactive aggression can be physical, but also verbal or relational and is strongly related to feelings of anger. High levels of reactive aggression are for instance reported in individuals with antisocial or borderline personality disorder as well as intermittent explosive disorder.

*Retaliation:* a harmful action against others in response to a real or perceived grievance. Typical triggers for retaliatory behavior are physical or social pain. Retaliation can be reinforced by the rewarding potency of revenge (5).

*Triggers for reactive aggression*

*Social threats:* interpersonal situations, such as invalidation, stigmatization, injustice, or rejection. In experimental paradigms, social threat is often simulated by angry facial expressions or in game tasks that imply negative treatment or punishment by another participant (6, 7). *Threat sensitivity* is an individual’s predisposition to direct attention to aversive stimuli and to respond to aversive cues on a behavioral and psychological level (8).

*Provocation:* an important cause of (reactive) aggression in humans. Provocations usually occurs in the form of unfair treatment and may be slights, insults, physical aggression, or impediment of personal goal attainment (1). Provocation mostly elicits negative feelings, such as anger and incites the provoked individual to act on it.

*Frustration:* a prevention of goal achievement (e.g., by omission of reward or frustrative non-reward) (9) or a state of emotional distress caused by failed achievement. Frustrative non-reward is related to reactions elicited by prevented or withdrawn reward.

*Regulatory functions/ functions of cognitive control*

*Cognitive reappraisal:* an emotion regulation strategy that modulates emotional reactivity by cognitively reevaluating and reframing emotionally arousing stimuli or situations, e.g., by thinking that a certain event only happened in a movie and not in real-life (10). Cognitive reappraisal has been regarded as an adaptive regulation strategy in times of low to moderate distress since it can effectively and lastingly reduce emotional arousal and reactivity.

*Cognitive distraction/distancing:* an emotion regulation strategy that aims at reducing emotional reactivity by shifting mental focus away from aversive stimuli or situations, ideally before the generation of negative emotions, e.g., by not looking at the worst or more threatening features of a scene. It is more effective in mildly to moderately distressing stimuli (11).

*Emotion regulation*: perception and recognition of one’s emotions and ability to modify them via automatic and effortful processes in a way that serves the attainment of one’s goals (12, 13).

*Inhibitory control:* an individual’s ability to deliberately suppress or override dominant thoughts, actions, perceptions or emotions. It is regarded as an executive function (14).

*Theory of mind:* ability to infer and interpret others’ mental states, emotions, and intentions (15).

*Aggression-inducing paradigms*

*Anger Articulated Thoughts during Simulated Situations (ATSS) paradigm:* During the ATSS paradigm, subjects are presented with audiotaped situations which elicit angry or happy emotions and are instructed to articulate their thoughts while trying to immerse themselves in these situations (16). In fMRI adapted versions of the paradigm, participants can be instructed to either focus on their emotions while trying to engage in the situation or to distract themselves from it (17).

*Inequality Game (IG):* an economic game in which participants face fictitious opponents who are programmed to behave either economically fair or unfair and to make encouraging or derogatory comments. The paradigm has different phases during which the participants either make economic choices and comments themselves or are subjected to the choices and comments of the programmed opponent (18).

*Point Subtraction Aggression Paradigm (PSAP):* a classical and frequently used experimental paradigm to induce and measure reactive aggression (19). In each trial, participants have to choose between pushing one of two or three buttons: button 1 for gaining points that may be exchanged in money, button 2 (aggression) to steal money from a fictitious opponent without gaining anything for themselves, and, in some variants, button 3 for protecting their account from attacks of their opponent. Aggression is provoked by their opponent stealing points from their account.

*Social Network Aggression Task (SNAT):* During the Social Network Aggression Task, participants are required to fill in a social network profile on which they receive negative, positive or neutral feedback by fictitious peers, represented on screen by photographs. Participants can in turn react to feedback by sending intensity modulated noise blasts to their “peers” in response to the feedback (20, 21).

*Social Threat Aggression Paradigm (STAP):* a modified version of the TAP, in which participants are presented brief videos displaying their opponent with either a neutral or angry facial expression before the punishment selection (22). This allows for a more direct investigation of facial threat processing within a hostile interpersonal encounter.

*Taylor Aggression Paradigm (TAP):* a widely used competitive reaction time task played against one or more fictitious opponents (23). Aggression is provoked by unfair treatments, i.e., high punishment settings of one opponent and is measured as the intensity of punishment set by the participant. In case of lost trials, participants are exposed to the punishment (i.e., an aversive event, such as an electric shock or a loud noise). Several modifications of the original paradigm are available, such as money subtraction as punishment or versions where the competitive encounter can be avoided completely for a limited number of trials (24).

*Ultimatum game (UG):* In the two-player Ultimatum Game (25), the first player (proposer) offers a split amount of pre-allocated money to the second player (responder), who in turn can decide to accept or decline the offer. When an offer is accepted, both players receive the proposed split of money, while no money is gained after declined offers. Aggression in this paradigm is defined by the number of declined offers and is provoked by unfair offers.

*Other tasks*

*Approach Avoidance tasks (AAT):* Approach-Avoidance tasks (26) measure the ability to override fast tendencies to approach appetitive and to avoid aversive stimuli. During socioemotional AATs, participants have to move their arm or a joystick towards or away from themselves in response to angry and happy facial expressions. Overriding fast behavioral tendencies is related to prolonged reaction times and activations in the ventrolateral prefrontal cortex that has been implicated in inhibiting fast amygdala-driven behavioral responses (27).

*Cyberball Paradigm:* a virtual ball tossing game with three or more players, has been designed to experimentally investigate social exclusion and its emotional, cognitive, and behavioral consequences (28). During the inclusion condition, participants receive an equal amount of ball tosses by their fictitious co-players, whereas in the exclusion condition they receive noticeably less or none at all. This has been found to induce negative feelings, such as anger and increases the likelihood for aggression (5).

*Go/No-Go tasks (GNGT):* Go/No-Go tasks (26) measure response inhibition and require participants to respond to a stream of stimuli with button presses to one type of stimuli (Go-trials) and withholding a response when another type of stimuli is presented (No-Go-trials).

**References**

1. Anderson CA, Bushman BJ. Human aggression. Annu Rev Psychol. 2002;53:27-51.

2. Coccaro EF. Intermittent explosive disorder: development of integrated research criteria for Diagnostic and Statistical Manual of Mental Disorders, Fifth Edition. Compr Psychiat. 2011;52:119-125.

3. Blanchard DC. Translating dynamic defense patterns from rodents to people. Neurosci Biobehav R. 2017;76:22-28.

4. Danesh HB. Anger and fear. The American journal of psychiatry. 1977;134:1109-1112.

5. Chester DS, Lynam DR, Milich R, DeWall CN. Neural mechanisms of the rejection-aggression link. Soc Cogn Affect Neurosci. 2018;13:501-512.

6. Rosen J, Kastrati G, Ahs F. Social, proximal and conditioned threat. Neurobiology of learning and memory. 2017;142:236-243.

7. Blair RJR. The Neurobiology of Impulsive Aggression. J Child Adol Psychop. 2016;26:4-9.

8. Carver CS. Threat sensitivity, incentive sensitivity, and the experience of relief. Journal of personality. 2009;77:125-138.

9. Berkowitz L. Frustration-aggression hypothesis: examination and reformulation. Psychological bulletin. 1989;106:59-73.

10. Gross JJ. Antecedent- and response-focused emotion regulation: Divergent consequences for experience, expression, and physiology. Journal of Personality and Social Psychology. 1998;74:224-237.

11. Gross JJ. Sharpening the focus: Emotion regulation, arousal, and social competence. Psychol Inq. 1998;9:287-290.

12. Gross JJ, Barrett LF. The emerging field of affective science. Emotion. 2013;13:997-998.

13. Thompson RA. Emotion regulation: a theme in search of definition. Monographs of the Society for Research in Child Development. 1994;59:25-52.

14. Stahl C, Voss A, Schmitz F, Nuszbaum M, Tuscher O, Lieb K, Klauer KC. Behavioral components of impulsivity. Journal of experimental psychology General. 2014;143:850-886.

15. Kanske P, Bockler A, Singer T. Models, Mechanisms and Moderators Dissociating Empathy and Theory of Mind. Curr Top Behav Neurosci. 2017;30:193-206.

16. Davison GC, Navarre SG, Vogel RS. The Articulated Thoughts in Simulated Situations Paradigm - a Think-Aloud Approach to Cognitive Assessment. Curr Dir Psychol Sci. 1995;4:29-33.

17. Tonnaer F, Siep N, van Zutphen L, Arntz A, Cima M. Anger provocation in violent offenders leads to emotion dysregulation. Sci Rep. 2017;7:3583.

18. Klimecki OM, Vuilleumier P, Sander D. The Impact of Emotions and Empathy-Related Traits on Punishment Behavior: Introduction and Validation of the Inequality Game. Plos One. 2016;11.

19. Cherek DR, Lane SD. Laboratory and psychometric measurements of impulsivity among violent and nonviolent female parolees. Biol Psychiat. 1999;46:273-280.

20. Somerville LH, Heatherton TF, Kelley WM. Anterior cingulate cortex responds differentially to expectancy violation and social rejection. Nat Neurosci. 2006;9:1007-1008.

21. Achterberg M, van Duijvenvoorde ACK, Bakermans-Kranenburg MJ, Crone EA. Control your anger! The neural basis of aggression regulation in response to negative social feedback. Soc Cogn Affect Neur. 2016;11:712-720.

22. Buades-Rotger M, Engelke C, Beyer F, Keevil BG, Brabant G, Kramer UM. Endogenous testosterone is associated with lower amygdala reactivity to angry faces and reduced aggressive behavior in healthy young women. Sci Rep. 2016;6:38538.

23. Taylor SP. Aggressive behavior and physiological arousal as a function of provocation and the tendency to inhibit aggression. J Pers. 1967;35:297-310.

24. Beyer F, Buades-Rotger M, Claes M, Kramer UM. Hit or Run: Exploring Aggressive and Avoidant Reactions to Interpersonal Provocation Using a Novel Fight-or-Escape Paradigm (FOE). Front Behav Neurosci. 2017;11:190.

25. Guth W, Schmittberger R, Schwarze B. An Experimental-Analysis of Ultimatum Bargaining. J Econ Behav Organ. 1982;3:367-388.

26. Donders FC. On Speed of Mental Processes. Acta Psychol. 1969;30:412-&.

27. Kaldewaij R, Koch SB, Volman I, Toni I, Roelofs K. On the Control of Social Approach-Avoidance Behavior: Neural and Endocrine Mechanisms. Curr Top Behav Neurosci. 2017;30:275-293.

28. Williams KD, Cheung CK, Choi W. Cyberostracism: effects of being ignored over the Internet. J Pers Soc Psychol. 2000;79:748-762.
